# Tumor Cell Infiltration into the Brain in Glioblastoma: From Mechanisms to Clinical Perspectives

**DOI:** 10.3390/cancers14020443

**Published:** 2022-01-17

**Authors:** Fidan Seker-Polat, Nareg Pinarbasi Degirmenci, Ihsan Solaroglu, Tugba Bagci-Onder

**Affiliations:** 1 Brain Cancer Research and Therapy Laboratory, Koç University School of Medicine, Istanbul 34450, Turkey; fseker14@ku.edu.tr (F.S.-P.); npinarbasi18@ku.edu.tr (N.P.D.); 2 Koç University Research Center for Translational Medicine, Istanbul 34450, Turkey; isolaroglu@ku.edu.tr; 3 Department of Neurosurgery, Koç University School of Medicine, Istanbul 34450, Turkey; 4 Department of Basic Sciences, Loma Linda University, Loma Linda, CA 92354, USA

**Keywords:** glioblastoma, invasion, dispersal, therapeutics, high-throughput screening

## Abstract

**Simple Summary:**

The estimated survival time for glioblastoma patients is extremely low; only about 5% of patients survive five years post diagnosis. The standard of care for glioblastoma patients involves surgery, radiation therapy, and chemotherapy with temozolomide. However, due to the extremely invasive capability of glioblastoma cells, tumors develop very diffusely, integrating into the healthy brain tissue. Indeed, the separation of healthy brain tissue and the tumor boundaries, by standard surgical microscopy, is very challenging. Therefore, the maximum safe removal of the tumor mass is difficult, leaving some tumor cells behind. Therefore, understanding the molecular mechanisms of tumor cell infiltration and developing anti-invasive approaches are of the utmost priority. Here, we provide a review of the characteristics and molecular mechanisms of glioblastoma invasion, and include a perspective of clinical applications.

**Abstract:**

Glioblastoma is the most common and malignant primary brain tumor, defined by its highly aggressive nature. Despite the advances in diagnostic and surgical techniques, and the development of novel therapies in the last decade, the prognosis for glioblastoma is still extremely poor. One major factor for the failure of existing therapeutic approaches is the highly invasive nature of glioblastomas. The extreme infiltrating capacity of tumor cells into the brain parenchyma makes complete surgical removal difficult; glioblastomas almost inevitably recur in a more therapy-resistant state, sometimes at distant sites in the brain. Therefore, there are major efforts to understand the molecular mechanisms underpinning glioblastoma invasion; however, there is no approved therapy directed against the invasive phenotype as of now. Here, we review the major molecular mechanisms of glioblastoma cell invasion, including the routes followed by glioblastoma cells, the interaction of tumor cells within the brain environment and the extracellular matrix components, and the roles of tumor cell adhesion and extracellular matrix remodeling. We also include a perspective of high-throughput approaches utilized to discover novel players for invasion and clinical targeting of invasive glioblastoma cells.

## 1. Introduction

Glioblastoma is the most common and malignant primary brain tumor [1]. Despite the advances in diagnosis and treatment, life expectancy still remains at approximately 12–18 months [2]. Invasion ability towards the surrounding tissue is a determinant for malignant tumor progression [3]. The high mortality of glioblastoma patients is partly attributed to the tumors’ therapy-resistant nature, as well as to the extremely invasive behavior of tumor cells. Extensive tumor cell infiltration (dispersal) from the primary tumor site into healthy adjacent tissue results in rapid and almost inevitable recurrence. Nearly all patients with glioblastoma, within a 2-year period from the time of primary surgery, experience tumor regrowth. Maximal safe tumor resection is important in terms of decreasing the tumor burden before radiotherapy and chemotherapy, and in reducing the risk of recurrence. However, the fact that healthy brain tissue and the boundaries of the tumor cannot be separated, either by eye or by standard surgical microcopy, makes the maximum safe removal difficult. Taking healthy tissue near the tumor environment during the removal of tumors located in functional areas of the brain or adjacent to the brain poses a risk for neurological sequelae.

**Figure 1:**
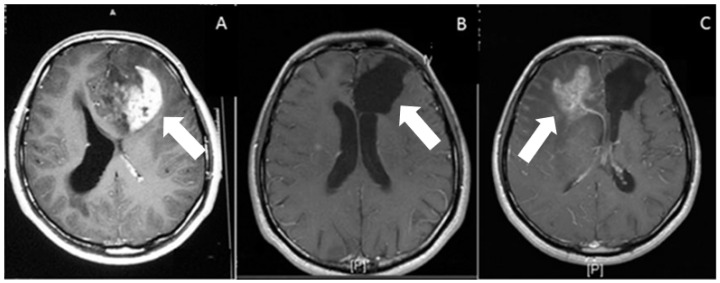
Post gadolinium contrast administration, T1-weighted axial images. (**A**) Preoperative, heterogeneous irregular enhancement, associated with the left frontal-lobe glioblastoma (arrow). (**B**) Postoperative (at 1 month) axial weighted image. On postoperative image, there is no residual enhancement. Arrow shows operation cavity. (**C**) Postoperative (at 18 months) axial weighted image shows recurrence of the tumor (white arrow) on contralateral hemisphere, associated with peripheral edema.

The current treatment for glioblastoma patients is the maximal safe tumor resection followed by the Stupp protocol, which is radiotherapy combined with concurrent daily temozolomide, followed by adjuvant temozolomide treatment [4]. However, the invasive nature of glioblastoma cells limits the therapeutic efficacy. The distant invasion of tumor cells to the brain parenchyma, and their infiltration and cooperation with healthy tissue, are major obstacles limiting complete tumor resection. Indeed, in glioblastomas, tumor borders are diffuse, and individual cells that have infiltrated into the healthy parenchyma are not detectable [5]. Therefore, the infiltrative cells that escape from surgery can recolonize and cause therapy-resistant recurrent tumor growth. Due to the diffuse nature of these tumors, radiotherapy applications can prove very challenging [6,7,8]. Figure 1 depicts the MRI scans of a glioblastoma patient before surgery, right after the surgical removal of tissue, and 18 months following surgery. Despite quite successful tumor resection and combined radiotherapy and chemotherapy, the tumor recurred in the contralateral hemisphere because of invasive cells infiltrating into the brain parenchyma, serving as an example and a testament to a huge clinical problem.

Despite the realization that the invasive nature of glioblastoma cells has drastic results for therapy resistance, high recurrence rates, and poor survival rates, there is no directed therapy to prevent this invasive behavior [5]. Therefore, understanding the mechanisms of glioblastoma cell invasiveness is of the utmost priority to develop successful therapeutic approaches. Here, we review the characteristics and molecular mechanisms of glioblastoma cell invasion. We also include a perspective on high-throughput approaches for invasion studies and clinical targeting of invasive cells.

## 2. Routes of Glioblastoma Cell Invasion

Invasive glioblastoma cells diffusely infiltrate into normal brain tissue, which limits the therapeutic options and increases recurrence rates by inducing the formation of secondary tumors. To interpret the mechanisms facilitating the glioblastoma’s invasive behavior, molecular mechanisms of tumor cell migration, migration routes, and accumulation sites of tumors cells in the brain need to be better understood. The preferred or avoided anatomical structures may affect the growth and invasion pattern of glioma tumors, giving rise to defined anatomical subtypes, such as the optic pathway or limbic gliomas [9,10,11].

**Figure 2:**
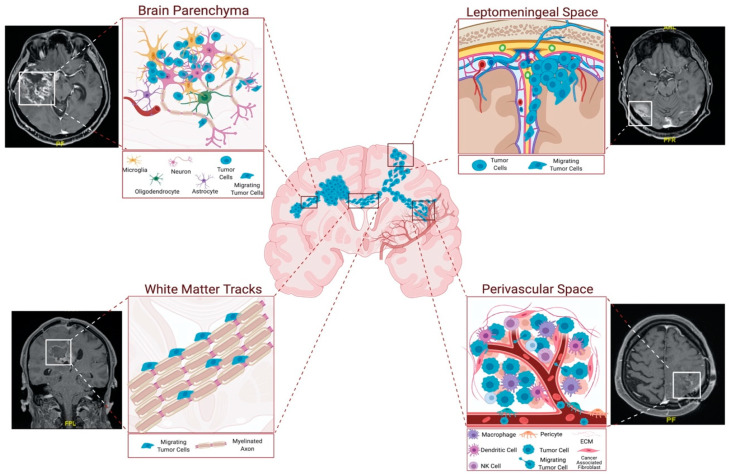
Routes of glioblastoma cell invasion. Glioblastoma cells generally invade using tracts in parenchyma, white-matter tracts, and leptomeningeal and perivascular spaces. Among these, perivascular space and white-matter tracts are the most preferred routes for glioblastoma invasion. Perivascular space attracts the tumor cells with the presence of blood vessels, which provide oxygen and nutrients. White-matter tracts are composed of myelinated axons, and tumor cells exploit these structures to reach distant locations in the brain. Parenchymal cells facilitate glioblastoma invasion by secreting several factors. Figure generated at Biorender.com, combined with representative MRI images from our clinic.

The invading cells tend to follow roadmaps that already exist in the brain. They co-opt and move along certain anatomical structures, such as perivascular space and white-matter tracts, and they can reach to distant sites in the brain parenchyma or the leptomeningeal space [12] (Figure 2). More specifically, invasive glioblastoma cells have a preference to move along myelinated fiber tracts, such as the corpus callosum and the internal capsule, meninges, ventricular lining, and basement membrane of blood vessels, subependymal space–perivascular regions, and glia limitans externa [13].

Since the vasculature and white-matter tracts are the primary structural components of the brain, these structures are the most preferred routes for glioblastoma cell invasion. The perivascular space surrounds blood vessels, including arteries, arterioles, and veins. Invasive tumor cells infiltrate the perivascular space to reach into the vessels, which provide oxygen and nutrients. Indeed, several studies have shown the significance of blood vessels as routes for invasive glioma cells [14]. Most importantly, it has been shown that over 85% of glioma cells move into contact with blood vessels when injected into the brain [15]. Furthermore, chemoattractants produced by endothelial cells can facilitate the recruitment of both invasive glioma cells and glioma stems cells to the perivascular space around blood vessels [15,16]. Invasion into the perivascular space disrupts the blood–brain barrier by separating the astrocyte end feet from the blood vessels [17].

The other primary invasion route, white-matter tracts, is composed of myelinated axons. The corpus callosum is the largest source of tracts, through which the tumor cells successfully invade and reach several distant brain locations [13]. Indeed, the invasive glioblastoma cells can exploit the connective utility of the corpus callosum or the anterior commissure, and can spread from one hemisphere to the other. Glioblastoma cells have been shown to enrich optic and pontine–white-matter structures, despite their distance from the primary site [18,19]. Indeed, these sites are well known to also harbor diffuse pediatric gliomas [20]. Cell movement along white-matter tracts is partly mediated by several axonal guidance molecules, such as proteins belonging to the slit, semaphorin, and netrin families [21].

Glioblastoma cells invade the brain parenchyma in small groups, and they interact with their environment in doing so [22]. There are a variety of cell types in the brain microenvironment, such as astrocytes, reactive astrocytes, oligodendrocytes, glial progenitors, microglia, glial progenitors, neural progenitors, neurons, neural stem cells, and vascular endothelium. In addition to the autocrine signals expressed by the tumor cells, all of these cell types secrete diverse ligands and mitogens that induce invasiveness in glioblastomas. The interactions between these cell types and the resulting changes in the microenvironment have important roles in glioma progression and invasion [23]. For example, higher invasion was observed when glioblastoma cells expressed EGFR and responded to EGF secretion from microglia [24]. Besides the important tumor cell–stromal cell interactions, the physical environment that the tumor cells encounter constitutes a major component in their invasive behavior. For example, the tumor cells disseminate passively within the perivascular space by the flow of cerebrospinal fluid and actively move along the ventricular lining, using cell-surface receptors [25,26].

The invasion of tumor cells also depends on the crosstalk between the invading cells and cues from the microenvironment. Hypoxia is one of the microenvironmental features affecting the invasive properties of glioblastoma cells. While tumors are growing rapidly, the need for an oxygen supply increases. Lack of oxygen causes intravascular thrombosis, hemorrhage, and eventually necrosis of the tumor tissue. To evade the hypoxic environment and to reach oxygen, tumor cells migrate away from the hypoxic area, produce angiogenic factors to induce blood vessel formation, and adopt anaerobic glycolysis [27].

## 3. Modes of Glioblastoma Invasion

Tumor cells may have different invasion mechanisms, depending on their association with other cells. They may migrate as individual cells or move together collectively. The extracellular matrix (ECM) composition, mechanical properties, such as stiffness or porosity, and topography are the factors that affect the migration and invasion properties of these cells [28]. At a single-cell level, invasion may be categorized as mesenchymal or amoeboid. While the amoeboid mode of invasion is based on the propulsive movement without proteolytic ECM remodeling, the mesenchymal mode involves focal interactions with ECM and movement in a traction-dependent manner, due to cytoskeletal contractility [29]. Due to the focal cell–ECM interactions, ECM-degrading proteolytic enzymes are recruited. These enzymes remodel ECM and generate a path for the invading cells [30,31].

**Figure 3:**
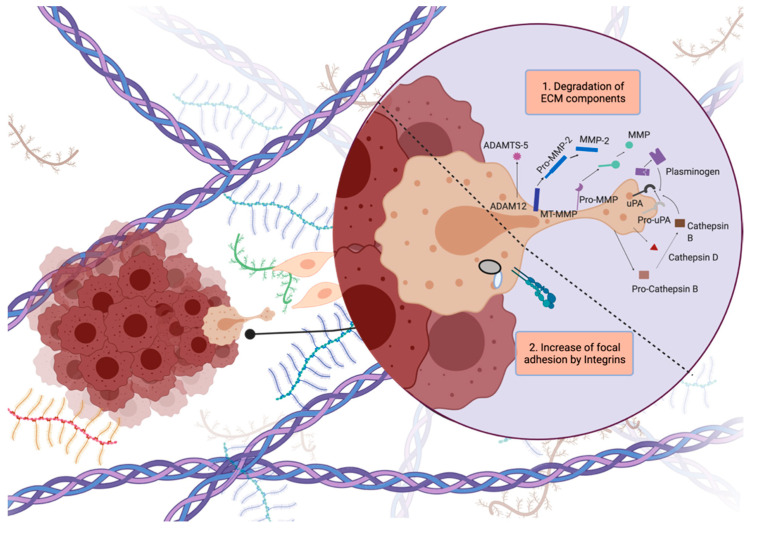
Steps of tumor cell invasion. Dynamic regulation of attachment–detachment cycles to break and generate contacts with ECM, and readjustment of cytoskeleton to generate protrusions are crucial for cell invasion. Figure generated at Biorender.com.

Glioblastoma cells display unique invasion features, as they invade locally inside the brain, instead of generating distant organ metastasis. Glioblastoma cells generally invade as single cells and they exhibit the mesenchymal mode of invasion, or so-called saltatory migration [32]. During invasion, tumor cells generate a strong adhesion force at the focal contacts on ECM by concentrating integrins. At the same time, they produce proteolytic enzymes such as matrix metalloproteinases (MMPs) to degrade the local ECM components by pulling and contracting the actin cytoskeleton to propel themselves toward the newly generated invasion path [5] (Figure 3).

In 3D collagen gels, a “leader” glioblastoma cell reorganizes the collagen and creates a track for invasive glioblastoma cells to follow [33]. These cells generate leading pseudopodia, which are short-lived, actin-rich membrane protrusions, and filopodia, which are long-lived, finger-like protrusions [34]. Generation of these extensions that explore the environment is thought to be the mechanism in vivo to move along the white-matter tracts and blood vessels. Formation of these protrusions is followed by the formation of focal adhesions at the front edge and dissolution of these adhesions at the rear edge of the cells to detach from these adhesions [35]. Dynamic regulation of attachment, protrusion, detachment, and readhesion is critical for controlling the cell movement. Strikingly, glioblastoma cells have been shown to generate an interconnected network in the brain and facilitate their infiltration using tumor microtubes, similar to their movement along other structures in the brain [36].

Recent efforts have been directed towards understanding the heterogeneity within tumors, focusing on the differences between cores and edges of glioblastomas. For example, in a recent mouse model established with regionally derived glioblastoma cells, it was demonstrated that cells from the edge formed highly invasive tumors, and cells from the cores formed more confined tumors with therapy resistance. Additionally, it was uncovered that the core and edge cells exhibited a paracrine crosstalk through soluble factors to maintain their phenotypes [37]. With the advanced imaging technologies, such as high resolution intravital microscopy of orthotopic mouse brain tumors, it has now been demonstrated that tumor cell dynamics vary at different locations [38]. In the study by Alieva et al., various behaviors of tumor edges have been defined, such as slow but directed motility, or fast but less directed motility. Taken together, better understanding of the routes, patterns, and modes of glioblastoma invasion will provide clues for designing more effective surgical and therapeutic interventions.

## 4. Molecular Mechanisms of Glioblastoma Cell Invasion

### 4.1. Role of Extracellular Matrix in Invasion

Tumor cell invasion is regulated by biophysical and biochemical stimuli received from the complex ECM networks [39]. Glioblastoma cells infiltrate into brain tissue by interacting with various brain microenvironment components. Regulation of attachment–detachment and dynamic remodeling of ECM are major drivers of cell invasion [5,40].

Healthy brain ECM mainly consists of non-protein-bound, space-filling hyaluronic acid, and different types of proteoglycans and glycoproteins, which organize the extracellular space [41]. The vascular and subpial basement membranes contain ECM proteins, collagen IV, laminin, fibronectin, and vitronectin. The basement membranes of glia limitans externa and subependymal space consist of collagen I, III, IV, fibronectin, laminin, and several proteoglycans [42]. In addition to parenchymal cells forming the ECM, the tumor cells greatly contribute to the ECM composition by generating and secreting their own molecules. For example, cultured glioblastoma cells can generate basement membrane components, such as Laminin [43], Vitronectin [44], Fibronectin [45,46], Tenascin C, Collagen I [45,47], Collagen IV [45,48], or Collagen VI [49]. While ECM composition is tightly controlled in physiological conditions, the loss of this control can contribute to invasion and metastasis. Similarly, the ability of cells to adhere to ECM and the proteolytic cleavage of ECM also change during tumor cell invasion [50,51].

Glioblastoma dissemination routes, such as myelinated axon fibers, and vascular basement membrane and externa contain a specific ECM composition, including fibrous proteins, such as Collagens, Fibronectin, Laminins, and Vitronectin [41,52]. Accordingly, Vitronectin, Fibronectin, and Tenascin C expression is correlated with increasing invasiveness [53] and a higher glioma grade [54,55]. ECM proteins, such as Hyaluronan, Tenascin C, Osteopontin, SPARC, and Laminin expression, are upregulated at the invasive edge of glioblastomas [43]; Vitronectin is expressed at the margins of gliomas and surrounds invaded tumor cells [54,56]; and Laminin is highly expressed at the borders between the tumor and normal brain tissue [57,58]. Taken together, the ECM composition is a critical contributing factor for glioblastoma invasion.

### 4.2. Role of Adhesion Proteins in Invasion: Attachment to and Detachment from ECM

Cells need to regulate their ECM interactions to coordinate their movement. In the case of tumor cell invasion, cells first detach from ECM, and invading cells reattach to ECM to retain a directional movement. Indeed, local detachment of tumor cells from the primary tumor and their interaction with the adjacent parenchymal tissues facilitate distant movement [39]. Tumor cell adhesion is enhanced in the ECM-rich regions of the brain, further supporting that ECM interactions and increased adhesion facilitate glioblastoma invasion [32]. The adhesive properties of tumor cells are significant determinants of their invasive potential, and many adhesion-related proteins have been proposed as potential targets to inhibit invasion [59].

Cells first need to detach from the tumor mass to invade. In this stage of invasion, cell adhesion molecules CD44, the neural cell adhesion molecule (NCAM), and cadherin proteins have been shown to have significant roles. CD44 is a transmembrane glycoprotein and an adhesion molecule interacting with hyaluronic acid. Since hyaluronic acid is a significant portion of the brain ECM, CD44 expression has a significant role in physiological and pathological cases [60]. While a variant of CD44 expression is detected in metastatic tumors [61], the cleaved CD44 is detected in 60% of gliomas [62], and its expression correlates with the glioma grade [63,64].

NCAM is a glycoprotein in the immunoglobulin receptor superfamily, and its role in ECM degradation has been previously shown [65]. In the case of glioblastoma invasion, NCAM acts as a paracrine inhibitor by interacting with the cell surface receptor and other NCAM-expressing cells. NCAM ectopic overexpression has been shown to decrease glioma cell motility [66].

Besides these, cadherins, which are calcium-dependent, transmembrane, cell-adhesion molecules and mediate cell-to-cell adhesion, have been shown to play important roles in glioblastoma invasion. E and N cadherins bind to β-catenins and, in turn, β-catenins bind to α-catenins, acting as bridges to bind the cadherin complex to the actin cytoskeleton. The switch from E-cadherin to N-cadherin has been associated with cell motility and a transition to a more invasive phenotype [67]. In glioblastoma, it has been shown that instability and disorganization of cadherin-mediated junctions increase invasion [68].

For the invading cells to reattach to ECM, the proteins Tenascin-C and Integrins have key roles. Tenascin-C is an ECM protein that induces filopodia formation, is overexpressed in gliomas [44,69], and is produced by the tumor cells [44]. Integrins are the most important class of adhesion molecules interacting with ECM proteins and other cell surface adhesion molecules, such as ICAM-1, ICAM-2, and VCAM-1. By facilitating these interactions, Integrins have a central role as bridges between extracellular contacts with the intracellular cytoskeleton [70]. Cells can only move from one site to another by forming and breaking Integrin-mediated ECM interactions [71]. In accordance with their important roles, various Integrin family members are upregulated in glioma cells [72,73,74]. In addition to promoting cell adhesion and migration, Integrins also have a role in intracellular signaling by transducing and coordinating the extracellular signals. Focal adhesion kinase (FAK), which is activated by Integrin-mediated ECM-adhesion and several growth factors, coordinates cytoskeleton rearrangement by recruiting cytoskeletal proteins and activating Rho GTPases [75]. Integrin clusters aggregate together with cytoskeletal proteins, such as vinculin and FAK, to generate adhesion complexes [76]. FAK is overexpressed in many types of cancers, including gliomas [77], and is known to promote cell migration [78]; its expression correlates with increased invasiveness and recurrence [79]. Glioblastoma cells have increased FAK expression at their invasive edges [80].

Taken together, attachment to and detachment from ECM through regulated interactions with Integrins constitute a critical component of the glioblastoma invasion mechanism.

### 4.3. Role of Proteinases in Invasion: Remodeling of ECM

Invasive tumor cells disrupt and remodel ECM components to generate a path to move and penetrate adjacent normal brain tissue. In addition to opening space for movement, active degradation also liberates growth factors and matrix proteins trapped in ECM, promoting invasion further [81]. Since proteolytic degradation of ECM is crucial for invasion, glioblastoma cells depend on proteinases and/or proteinase activators, such as matrix metalloproteinases (MMP), the plasminogen activator (PA) system, and cathepsins.

*MMPs:* MMP activity is shown to be significant for invasion because MMP-2, MMP-9, and MT-MMPs can together degrade almost all types of ECM; they are specifically activated in tumor tissues, and their activation correlates with a poor prognosis and invasion [82]. MMP2 activity is highly increased in glioblastoma tumors, compared to normal brain tissue, and expression levels correlate with malignant progression [83,84]. MMP9 expression is generally detected at tumor margins and at the endothelial proliferation sites, which relates its expression with angiogenesis and invasion [85]. Inhibition of MMP2 or MMP9 activity, or downregulation of MMP2 and MMP9 protein levels, have been reported to decrease invasion in cell lines and mice models [83,84].

Similarly, the ADAM family contains metalloproteinase domains homologous to MMPs. ADAM12m and ADAMTS-5 are overexpressed in glioblastoma [86,87].

*Plasminogen Activator System*: The PA system is a key player in ECM remodeling. Active plasmin generated by this system degrades ECM directly or indirectly via the activation of MMPs [88], and facilitates cell migration and invasion in the context of cancer. This focal proteolysis carried out by the PA system reorganizes ECM; changes cell–ECM interactions via Integrin receptors, and releases mitogenic and motogenic molecules from the matrix [89]. The central tumor-promoting role of plasminogen activator inhibitor-1 (PAI-1, also known as SERPINE1), which is a member of the PA system in cell migration, cancer invasion, and tumor vascularization, has been shown in several cancer types [90]. In glioblastoma, overexpression of PAI-1 has been shown to correlate with a poor prognosis and reduced survival [91], and PAI-1 plasma level is a predictive marker of the glioma grade [92].

*Cathepsins:* Cathepsin is lysosomal acid hydrolase, which has a role in protein degradation in lysosomes. In the context of cell migration, it has been shown to participate in localized degradation of ECM proteins by activating MMPs and uPA [93,94,95]. Cathepsin B is overexpressed during glioma cell migration and its expression correlates with the glioma grade [96]. Together, proteinases and their ECM degradation activities are major players in glioblastoma invasion.

### 4.4. Cytoskeletal Changes during Invasion

Cytoskeleton rearrangement is crucial for the movement of the cells. During their movement, tumor cells become polarized with leading and trailing edges, and they generate protrusions that facilitate their movement. For cells to move forward, the cytoskeleton should be rearranged with assembly, disassembly, and organization. To this end, a large degree of actin polymerization followed by depolymerization is needed. While these rearrangements generate protrusions from the cells to facilitate cell movement, microtubules and intermediate filaments maintain the cell structure and hold the organelles in place during this movement [97]. Protrusion formation and actin polymerization is carried out by the Arp2/3 complex; an Arp2/3 complex-activating, nucleation-promoting factor; a barbed-end capping protein; and cofilin and profilin to bind actin monomers [98]. Lamellipodia and filopodia are actin membrane protrusions that drive cell migration. Rac1 and Cdc42 regulate their formation through several targets, including Pac1, and increase cell motility [99]. In the case of glioblastoma invasion, protrusions from the cell body called podosomes and invadopodia are crucial. These structures release high amounts of matrix-degradation enzymes and form the leading structure for invasion [100]. Invadopodia and podosome formation depend on the activity of cortactin, which is an actin-binding protein, and is induced by WASP/N-WASP activity [101]. In glioma cells, RhoA and Rac are the main molecules that regulate the cytoskeletal rearrangements that facilitate migration and invasion of the cells [102].

### 4.5. Role of Other Motility Factors in Invasion

Motility factors, which operate through autocrine and/or paracrine signaling, have significant roles for invasion. In the context of glioblastoma, invasion, scatter factor/hepatocyte growth factor (SF/HGF), and epidermal growth factor receptor (EGFR) act on cell motility. SF/HGF and its receptor c-Met, which is a proto-oncogene product and has tyrosine kinase activity, promote cell motility in glioblastoma [103]. EGFR amplification and overexpression are two of the most frequently seen genetic alterations in glioblastoma [104]. Amplification of EGFRvIII, the constitutively active, truncated form of EGFR, is associated with a poor prognosis, since EGFR signaling enhances proliferation, migration, and invasion of glioblastoma cells [105,106]. Recently, microenvironment-related effects of the release of soluble ligands were described. For example, EGFR ligand amphiregulin from glioma-associated microglia stimulated glioma invasion [107].

There are other classes of molecules involved in glioblastoma invasion, such as secreted proteins such as the insulin-like growth-factor-binding protein family (IGFBPs), cysteine-rich 61/connective tissue growth factor/nephroblastoma overexpressed (CCN) family (Cyr61), angiopoietin 2 (Ang2), YKL40, and autotaxin (ATX)/lysophospholipase D. There are also membrane-type proteins, such as Fn14/TWEAK, EphB2/ephrin-B3, and CD155, associated with invasion. Especially, Ephrins and Ephrin receptors have an important role in bidirectional signaling in glioblastoma invasion. Ephrins mediate cell-to-cell signaling and control tissue organization, and the Ephrin pathway is overactivated in invasive glioblastoma cells [34]. Overactivation of Ephrin pathway elements, especially EphA2 and EphA3, has been associated with invasiveness and poor patient outcomes [34,68]. It was recently shown that TGFβ is associated with microtube formation and invasion in glioma cells through SMAD and Tsp1 signaling, as microtube formation is one of the drivers of invasion [108]. The Rho family of GTPases from the Ras superfamily, Rho-A, Rac1, and Cdc42, are responsible for the spatial regulation of glioblastoma invasion. They control actin-mediated cytoskeleton rearrangements that facilitate cell movement [109]. In glioblastoma, the increased activity of Rac1, Cdc42, RhoA, and RhoG is associated with the invasiveness of tumor cells [110,111,112]. Pleckstrin homology domain-interacting proteins (PHIP) have previously been shown to be potential targets for pancreatic and several other different cancer types; a very recent study showed that PHIP also acted on the focal adhesion complex and drove glioblastoma invasion [113]. TRIM28, which is a transcription factor involved in gene regulation, is also involved in the proliferation of tumor cells. Recently, in the study by Turnsek et al., TRIM28 was found to be enriched in the tumor core, and was associated with glioma cell invasion in a zebrafish model [114]. Additionally, recent studies showed that chemokine receptor type 4 (CXCR4) and C-X-C motif chemokine ligand 12 (CXCL12) promoted glioma cells to acquire more invasive phenotypes [115,116]. Together, the evergrowing list of molecules with functional roles in invasion is extensive; targeting these molecules or pathways for clinical translation is a priority in glioblastoma research.

## 5. Role of Ion Channels in Glioma Invasion

Tumor invasion is orchestrated not only by ECM modifications and cytoskeletal changes, but also in the regulation of ion channels. The activity of ion channels is crucial for cell homeostasis, resting membrane potential, regulation of cell volume in cellular physiology and proliferation, survival, and regulation of gene expression in cancer cells [117]. To protrude, glioma cells modify their ionic equilibrium, and adjust their volume to navigate the confined space. This ionic equilibrium is facilitated by the flux of various ions by ion channels and release of cytoplasmic water, ultimately causing the shrinkage of glioma cells. These volumetric adjustments are facilitated by differentiated ion channel activity on both the leading edge and the trailing edge of a protruding glioma cell [118]. The Ca^2+^ activated K^+^ channel KCa1.1 (KCNMA1) and Na^+^ K^+^ Cl^−^ cotransporter NKCC1 are known ion channels positioned on the migrating edge of glioma cells to facilitate migration via ion flux [118,119]. In addition, on the trailing edge of migrating glioma cells, the well-studied Ca^2+^ activated K^+^ channel KCa3.1 (KCCN4) and the voltage-gated Cl^−^ channel 3 CLC3 regulates the flux of K^+^ and Cl^−^ ions, causing a change in the cell volume [120,121]. In line with their mentioned roles, such ion channels facilitating migration are predominantly expressed in glioblastoma. In contrast, expression of Kir4.1, an inward rectifier K^+^ channel, is negatively correlated with the invasive behavior of glioma, as inhibition of Kir4.1 increases the migration capacity of glioma cells [122]. The ion channels’ roles in invasion are not confined to their effects on volumetric regulation. For example, the flux of ions such as Ca^2+^ and Cl^−^ results in activation of downstream signaling pathways, such as the NF-KB pathway (TMEM16A), Ca^2+^-dependent pathways, and STAT3 and Notch signaling pathways via the transient receptor potential (TRP) family member TRP-melastatin 7 (TRPM7) [123,124,125]. Accordingly, it has been shown that pharmacological inhibition of TRPM7 reduces the invasion capacity of U87 cells [126]. Lastly, it has been reported that acid-sensing ion channels are also overexpressed in glioma cells, which produces acidosis as the tumor cells migrate [127].

## 6. Epithelial-to-Mesenchymal Transition and Invasion

Epithelial-to-mesenchymal transition (EMT) is a process during which an epithelial cell undergoes substantial transcriptional, biochemical, and structural changes, resulting in the adoption of a mesenchymal cell phenotype [128]. Accordingly, cells that have acquired mesenchymal characteristics are more migratory, exhibit more resistance to apoptosis, and they produce pro-invasive factors that facilitate invasion in the neighboring tissue. Therefore, EMT has an important role in cancer progression by controlling the transcriptional programs operating during the transition between tumor growth and metastasis. This transition brings along the changes in the gene expression and functional behavior, which affect the motility phenotype. Roughly, cell–cell and cell–ECM interactions are changed and established, the cytoskeleton is reorganized, and transcription of the genes that support the invasive mesenchymal phenotype is activated [129]. While epithelial cells are anchored to the basement membrane with tight interactions, the attachments to ECM with focal adhesion molecules loosen when the cells undergo mesenchymal transition [130]. In the context of cancer, EMT is linked to enhanced motility, chemoresistance, and stem cell-like properties [131]. Proliferative tumors generally exhibit epithelial-like morphology with tight cell junctions and E-cadherin overexpression on the cell membrane. When these cells undergo EMT, the cells become more fibroblast-like, tight junctions disappear, and E-cadherin expression is majorly lost. The cells that undergo EMT degrade the ECM and become more invasive. With this increased invasiveness, they can enter circulation (intravasation) easily and survivors can leave the blood or lymphatic vessels (extravasation). After extravasation, the cells reverse the EMT program and undergo mesenchymal-to-epithelial transition (MET) [132]. This is a strategy for cancer cells to metastasize and generate secondary tumors in distant areas [27].

While EMT is a known driver of invasion and metastasis in mainly carcinomas, EMT-like processes have been described to participate in the progression of gliomas, as well [133]. Invasive gliomas share common molecular features with metastatic epithelial cancers [134]. ZEB1 transcription factor, which is a key regulator of EMT, is also a driver of glioblastoma invasion [135]. Overexpression of Decorin, a member of the small leucine-rich proteoglycans (SLRPs), is associated with a decreased EMT-like phenotype and suppressed invasion ability in glioma cells [136]. Twist overexpression has been shown to promote glioma invasion [137]. Silencing of Snail in glioma cells has been shown to reduce invasion [138], and Slug expression has been shown to correlate with histological grade and increased invasiveness [139]. Recently, it was demonstrated that prolyl-4-hydroxylase subunit 2 (P4HA2), which is involved in collagen biogenesis, was one of the key players in the tumor microenvironment, and that P4HA2 promoted EMT, cell proliferation, and invasion in glioblastoma [140,141]. Other factors, such as TGFβ, TNFα, IL6, HGF, EGF, and PDGF are also regulators and inducers of EMT and are all implicated in glioblastomas [27,142,143].

## 7. Unbiased, High-Throughput Experimental Approaches to Study Glioblastoma Invasion

As mentioned, the aggressive and invasive nature of the glioblastoma cells are among the main reasons for therapy resistance, high recurrence, and eventually a poor prognosis. With this notion, several studies employed high-throughput strategies to identify and study novel molecules implicated in glioblastoma invasion (Table 1).

Mariani et al. used human glioblastoma tumor specimens and separated the cells residing in the core and the invasive rim by laser capture microdissection. When mRNA expression profiles of these cells were compared, they identified that the *P311* gene was upregulated in invasive cells, and this gene had a potential role in glioma invasion [144]. Hoelzinger et al. conducted a microarray analysis of the cells from patient tumor cores and the cells that invaded the white matter, using laser-capture microdissection. Besides several other genes, they found that *ATX* and *BCLW* were upregulated in invasive cells [145]. Demuth et al. utilized a three-dimensional invasion system, where glioma cell-line spheroids invaded collagen. With microarray analysis, expression profiles of core and rim cells were determined, and they reported that MKK3 and its downstream effector p38 were drivers of glioma invasion [146]. Again, Demuth et al. conducted a microarray screen to identify gene signatures in stationary and migratory populations. In this study, both cell lines and primary cultures were used; and migratory cells were separated from stationary cells using a radial migration assay. With the analysis of differentially expressed genes, they found 22 gene signatures, classifying glioblastoma cultures based on migration rate [147]. Recently, Krieger et al. conducted a single-cell RNA sequencing analysis and identified gene expression changes induced by the interaction between tumor cells and normal brain cells with a different experimental approach. They analyzed the changes in the transcriptional program of glioblastoma cells before and after coculture with iPSC-derived human cerebral organoids, which function as a scaffold for glioblastoma cell invasion [148].

**Table 1:** Molecules identified as having a role in glioblastoma invasion by unbiased, high-throughput approaches.

Approach	Gene/Protein Identified	Reference
Differential expression analysis of tumor cells from tumor core and the invasive rim by laser-capture microdissection	*P311*	[144]
Microarray analysis of the cells from tumor cores and the cells that invaded White matter using laser-capture microdissection	*ATX* and *BCLW*	[145]
Microarray analysis of core and rim cells using cell-line spheroids invading collagen	*MKK3* and *p38*	[146]
Microarray analysis of cell lines and primary cultures with radial migration assay	*CTGF*	[147]
RNA sequencing of motile and nonmotile cells using a spheroid dispersal model	*SERPINE1*	[149]
Differential expression analysis of long noncoding RNAs in glioma tissues, compared to normal brain tissues	*NEAT1*	[150]
miRNA profiling of slow-growing, diffusely infiltrating glioma and noninvasive primitive neural tumors	*miRNA-449a*	[151]
Functional screen with monoclonal antibody library generated against primary glioblastoma cells	*Itga7*	[152]
Analysis of enriched proteins on the cell membranes with different invasive capacities	*Itga5*, *CD97* and *Anxa1*	[153]
Analysis of proteins in cell lines with different invasive capacities	*Cathepsin D*	[154]
Functional proteomics approach with fluorophore-assisted light inactivation	*Neuropilin-1* and *Semaphorin3A*	[155]
Functional proteomics approach with fluorophore-assisted light inactivation	*CD155/PVR*	[156]
Analysis of proteins from glioblastoma sections by microdissecting cells from invasive border and proliferative core	*ELMO1* and *Dock180*	[157]
Proteomics analysis of xenograft models generated by serial transplantation of human glioblastoma specimens into rat brains	*PDI*	[158]

Another expression-profiling analysis was conducted by Zhou et al. to investigate differential expression of long noncoding RNAs in glioma tissues, compared to normal brain tissues. With this analysis, they found that lncRNA *NEAT1* inhibited glioma invasion by suppressing the expression of miR132, which regulates *SOX2* expression [150]. In addition, He et al. identified long noncoding RNA SLC8A1-AS1 as a regulator of glioma cell invasion through the Wnt-β-catenin pathway [159]. Li et al. conducted an miRNA screen in two distinct types of brain tumors; slow-growing, diffusely infiltrating glioma and noninvasive primitive neural tumors, and identified miRNA-449a as a suppressor of migration [151]. Another study by Diao et al. showed the role of miR-129-5p in glioma progression and invasion [160]. Xu et al. demonstrated lnc-NLC1-C associated with the low invasion and migration abilities of glioma cells through regulation of PRDX-3 expression [161]. Lin et al. showed that upregulation of the RNA-binding motif protein 8A (RBM8A) is associated with a poor prognosis in glioblastoma, and its pro-oncogenic function is facilitated through the Notch/STAT3 pathway [162].

Using an unbiased–functional proteomics approach called fluorophore-assisted light inactivation (FALI), Bagci et al. identified Neuropilin-1 and its ligand Semaphorin3A as mediators of glioblastoma invasion [155]. Previously, with a similar approach, Sloan et al. showed the role of CD155 as a mediator of glioma cell migration [156]. Goplen et al. obtained two different glioblastoma tumor types by serial transplantation of human glioblastoma biopsies into rat brains. While the tumors were very invasive at first transplantation, they switched to a less invasive phenotype by a transplantation series. They analyzed these two types of xenografts with a global proteomics approach and found that PDI was a significant player in glioma cell invasion [158]. Jarzynka et al. used glioblastoma sections to separately microdissect cells from the invasive border and the proliferative core to extract proteins from these populations. With this study, they identified ELMO1 and Dock180 levels were higher in invading cells [157]. Formolo et al. analyzed the secretome profiles of glioblastoma cell lines and demonstrated a correlation between secreted proteins and the invasive behavior of the cells [163]. Pei et al. used two different cell lines with different invasive properties and analyzed the proteins that may contribute to this behavior. They found that Cathepsin D might have a role in glioma cell invasion [154]. Duthika et al. compared the invasiveness of glioblastoma cell lines based on their invadopodia formation, and analyzed the enriched proteins on the membranes of these different cell lines. Among the proteins correlated with cell invasiveness, Itga5, CD97, and Anxa1, had prognostic values that could be used as therapeutic targets [153]. With a different approach, Haas et al. conducted a screen for a monoclonal antibody library generated against primary glioblastoma cells. This screen identified Itga7 as a functional marker of glioblastoma stem cells and the roles of Itga7 in tumor growth and invasion were validated with functional experiments [152]. Recently, Yong et al. showed that fibrinogen could be a mediator for brain tumor-initiating cells (BTICs), which may be a target for therapy to reduce BTIC tumorigenicity [164].

**Figure 4:**
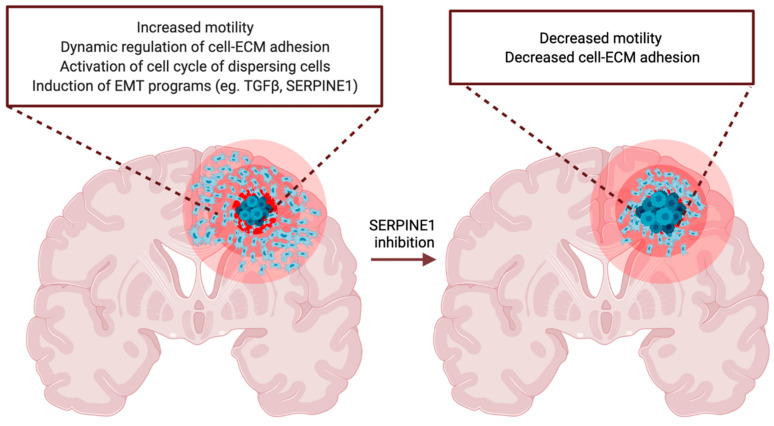
Effect of SERPINE1 on glioblastoma cell invasion. Transcriptome profiling of motile and nonmotile glioblastoma cells identified SERPINE1 as a regulator of glioblastoma cell motility. Inhibition or knock-down of SERPINE1 reduces glioblastoma cell invasion by regulating cell adhesion and directional persistence of the cells. As a result, SERPINE1 inhibition has the potential to reduce tumor progression in vivo. Figure generated at Biorender.com (adapted from Seker, F., et al. [149]).

Recently, we analyzed the dynamic changes in the transcriptome of motile and nonmotile glioblastoma cells in an unbiased manner [149]. Consistent with the former studies, we identified a large number of differentially expressed genes, most of which were upregulated during dispersal, revealing the dynamic and adaptable transcriptome of moving cells. We showed that *SERPINE1* gene expression is dramatically induced in cells on the move. Furthermore, we showed that SERPINE1 acts as a modulator of glioblastoma cell dispersal by regulating cell–substrate adhesion and directional movement of the cells (Figure 4).

All of these exploratory studies were conducted using different screen methods, techniques, and cell/tissue types, but ultimately with similar purposes. Even though these studies provide an essential insight for molecular mechanisms behind the movement of cancer cells, they are all limited in several aspects. First, several reports repeatedly showed that the cell lines hardly mimicked the in vivo conditions. The findings obtained with cell-line studies would be poorly applicable in in vivo conditions, since cell movement and cancer cell invasion highly depend on the cues from the native microenvironment [165]. To get a snapshot of the physiological conditions, the use of tissue samples would be a better solution. However, in the case of invasive glioblastoma, the tumor border is unclear, and the tumor is very infiltrative in nature. As a result, the separation of the tumor and healthy tissue, and the collection of samples prove challenging and remain limited by the anatomy and neurological function of the brain. To overcome this issue in the clinic, fluorescent-guided surgeries were performed to achieve the gross resection of tumors [166]. Moreover, by using Raman scattering histology, the intraoperative detection of residual tumor cells is now possible in a rapid and label-free manner [167]. Another limitation is the methods and techniques used in the studies. Conducting screens by using limited libraries restricts novel findings. To this end, conducting unbiased, genome-wide screens will open new ways for discovering novel molecular players of glioblastoma invasion. Adding to the complexity of glioblastomas, it is now well established that tumors display intratumor heterogeneity. Therefore, new advanced approaches to decipher the molecular mechanisms at a single-cell resolution level would be extremely informative in the future. For example, seminal works by Castro et al., or Patel et al. investigating the transcriptomic landscape of glioblastoma tumors at a single-cell level, provide insight about tumor heterogeneity and emphasize the prognostic value of such high-resolution approaches [168,169].

## 8. Clinical Targeting of Glioblastoma: Is There an Anti-Invasion/Anti-Migration Treatment?

*Standard of care:* The conventional care for glioblastoma involves tumor resection followed by radiation therapy and chemotherapy with TMZ [4]. TMZ (Temodar; FDA approval: 1997) is a DNA-alkylating agent that delivers a methyl group on specific sites of purine bases of DNA at the O6 and N7 positions of guanine and the N position of adenine [170]. O6 methylguanine is the main cause of TMZ toxicity, causing mismatches and, ultimately, DNA double-strand breaks, cell-cycle arrest at the G2/M phase, and apoptosis [171,172,173]. The differential response of patients to TMZ therapy and radiotherapy is linked to the activity of O6-methylguanidine DNA methyltransferase (MGMT), which is a DNA-repair enzyme and is regulated by promoter methylation. Patients whose tumor cells have a methylated MGMT promoter have a better response to TMZ treatment and have better overall survival. This makes MGMT promoter methylation both a predictive factor for TMZ response and a prognostic factor for survival [174]. Other than TMZ, two other FDA-approved oncology drugs are used for glioblastoma treatment: biodegradable carmustine and BCNU wafers (Gliadel; FDA approval: 1995). Even though these standard therapies increase the median overall survival time of patients, the tumors recur in nearly all cases [27]. Bevacizumab, which is an antiangiogenic agent by its ability to inhibit VEGF, has also been approved for use in glioblastoma patients (Avastin; FDA approval: 2009 for recurrent glioblastoma). However, trials have shown that treatment with Bevacizumab prolongs progression-free survival, but does not have any significant effect on overall survival [175].

A very important treatment approach for glioblastoma is radiation therapy. Currently, the European Organization of Research and Treatment of Cancer recommends a single-phase radiation therapy technique with a unique gross tumor volume, which comprises the T1 contrast enhancement region plus a margin of 20 to 30 mm, with a total dose of 60 Gy in 30 fractions [176]. During the radiation therapy, attention must be paid to ensure that the dose to critical structures (such as the brain stem, optic chiasm, optic nerves) is kept within acceptable limits [177]. However, a better understanding of glioblastoma migration and invasion patterns, and the ability to model the tumor distribution for each patient could allow for tailored radiation therapy maps in the future. For this reason, comprehending the aggressive and complex nature of glioblastoma invasion is an urgent unmet need [178].

Even though chemotherapy approaches seem to be useful at first treatment, tumor cells may acquire characteristics and may no longer respond to therapies [179]. To this end, the use of combinatorial chemotherapy approaches to overcome chemoresistance is an emerging and promising approach. Similarly, several innovations in radiotherapy are focused on increasing radiosensitization [180,181,182]. If successful anti-invasive strategies can be developed, their application together with chemo- or radiotherapy may halt the progression of glioblastomas and, ultimately, prevent recurrence.

*Immune Modulators:* Immune checkpoint inhibitors, especially the ones targeting PD-1 and CTLA-4, are currently being examined in clinical trials for glioblastoma. CAR-T cell applications, constructed based on patients’ tumor antigen profiles, are other immunotherapy approaches in clinical trials. Dendritic cell therapy is another personalized medicine application, which aims to target multiple glioblastoma antigens by using tumor lysates, RNA, peptides, and products of cancer stem cells. Vaccination with neoantigens derived from tumor-specific protein-coding mutations is another immunological strategy. The Gliovac vaccine is a promising treatment option in clinical trials [183]. Virotherapy uses genetically engineered oncolytic viruses to target glioblastoma [184].

*Targeted delivery approaches*: Current research in glioblastoma has evolved from focusing on only the therapeutic efficacy of new candidate drugs, to considering their delivery methods also. For example, even though a therapeutic drug can show efficacy in vitro, delivery of this drug to the tumor site through the blood–brain barrier (BBB) is among the biggest challenges in glioblastoma [185]. To this end, several drug-delivery methods are being developed with the help of the biomaterial sciences. Conjugated drugs, liposomes, monoclonal antibody-conjugated nanoparticles, nanotubes, and several other strategies are in development for efficient BBB penetration and specific recognition and targeting of glioblastoma tumors [186,187]. Another strategy is to use neural stem cells (NSCs) loaded with engineered viruses to increase targeted efficiency [178]. Similarly, NSCs secreting proapoptotic ligands that travel to the tumor site and deliver on-site therapies is a promising and emerging targeted approach [188].

*Tumor-treating fields*: Tumor-treating fields (TTFs) are a new and noninvasive treatment option currently approved and applied in the glioblastoma [189,190,191]. The treatment is delivered by electrodes attached to the scalp of the patient, which generate alternative electric fields that target the tumor [192,193]. The working mechanism of the TTFs is to lead the cancer cells to apoptosis by interfering with the mitotic spindle formation during cell division and damaging the cell structure [194]. While TTFs affect the cell division, the nondividing cells remain undisturbed, which makes TTFs a cancer-specific application [195]. The combination of TTFs with the conventional chemotherapy and radiotherapy has proven to be highly efficacious without severe side effects [192,196,197]. Since TTFs are a regional therapy with the delivery of the electric fields to a limited location, this option has been a promising one for glioma treatment, considering the diffuse infiltrative, but almost never metastasizing, nature of gliomas [198]. Even the parameters of TTF application should be optimized for each glioma subtype. The combination of TTFs with the standard therapy is a promising option for both newly diagnosed and recurrent glioma patients.

*Anti-invasive approaches:* Clearly, most therapeutics applied in the clinics for glioblastoma patients are directed towards halting tumor cell proliferation and inducing tumor cell death. Despite the fact that the invasive nature of glioblastoma cells has drastic results on therapy resistance and very high recurrence rates, there is no directed anti-invasion therapy approved for glioblastoma [5], except for ongoing clinical trials. Moreover, studies showed that current therapeutic strategies, such as Bevacizumab treatment, may further increase the invasiveness of the cells [199]. In some cases, resection has been shown to increase tumor malignancy through a stem cell repopulation effect. While chemotherapy causes further variations and mutations, low-dose radiation may increase the invasiveness of glioma cells [200,201].

The above-mentioned invasion pathways and signaling networks offer druggable targets, such as TGFβR1, Ephrin receptors, FAK, ROCK, CK2, AKT, JAK, NF-κB, STAT3, and EZH2. Among these, using inhibitors of TGFβR1, Ephrin receptors, FAK, ROCK, and CK2 would more specifically target invasive glioblastoma cells, sparing normal cells. The TGFβ inhibitor Galunisertib is in clinical development and is being tested against glioblastoma (clinicaltrials.gov identifier: NCT01682187 and NCT01582269) [202]. In a recently completed clinical trial (NCT01220271), it was demonstrated that a combination treatment of Galunisertib with temozolomide-based radiochemotherapy (TMZ/RTX) does not produce a significant difference in terms of efficacy, safety, or pharmacokinetic variables compared to radiochemotherapy alone [203]. For Ephrin receptors, a Phase I study is investigating KB004, a monoclonal antibody targeting EphA3 in glioblastoma (NCT03374943). FAK inhibitors have not been tested in glioma yet, but Phase II trials are ongoing for Defactinib and GSK2256098 (NCT01951690 and NCT02523014) [13]. Other than these trials, studies targeting glutamate signaling and ion receptors are ongoing [204]. A phase II clinical trial on recurrent glioblastoma patients showed a significant increase in the overall survival, although with more adverse effects (NCT01480479) [205]. Onartuzumab is a humanized monoclonal anti-MET pathway drug, and it has been in phase II clinical trials for recurrent glioblastoma patients. Even though the results showed no significant benefit, molecular analyses revealed that patients with a high expression of HGF or patients with unmethylated *MGMT* promoter were more likely to benefit from an Onartuzumab and Bevacizumab combination, leading to improved survival [206].

In addition to these ongoing trials, other elements linked to invasion have already been tested. The targeting of MMPs to reduce glioblastoma invasion evoked severe normal tissue side effects in patients and did not improve patient survival when combined with temozolomide [206]. The targeting of Integrins was more promising, but unfortunately, the patients with newly diagnosed glioblastoma and methylated *MGMT* promoter did not benefit from treatment [207]. Even though the targeting of proteases restricted the dissemination of tumors inside the brain, this approach did not improve overall patient survival [206].

## 9. Concluding Remarks

Given the aggressive characteristic of glioblastoma, the invasive glioblastoma cells are found already disseminated and embedded into the brain at the time of diagnosis. Despite tumor removal, these cells can recolonize and cause a recurrence of the tumor by resisting the conventional therapy approaches. Considering that conventional therapy approaches have a very limited effect on invasive glioma cells or cause adverse effects on healthy cells, understanding the mechanisms of glioblastoma cell invasiveness is important to develop effective therapeutic approaches. To this end, studies identifying druggable targets of glioblastoma invasion have a great potential to offer new approaches.

Therapeutics targeting invasion mechanisms would be useful to eradicate the population left behind after surgical removal and would make the tumors more manageable in the clinic, especially with complementary therapies that target other features of glioblastoma. However, considering that highly invasive cells have already disseminated to locations other than the primary tumor area and formed tumor seeds in secondary locations, preventing only the invasion may not eradicate these cells and may not change the patient’s survival considerably. To this end, anti-invasive therapies should be combined with other treatments. Offering such combinations would ultimately result in more manageable tumors and provide opportunities for better a prognosis for glioblastoma patients.

## References

[1] Ramirez Y.P., Weatherbee J.L., Wheelhouse R.T., Ross A.H. (2013). Glioblastoma multiforme therapy and mechanisms of resistance. Pharmaceuticals.

[2] Verhaak R.G.W., Hoadley K.A., Purdom E., Wang V., Qi Y., Wilkerson M.D., Miller C.R., Ding L., Golub T., Mesirov J.P. (2010). Integrated Genomic Analysis Identifies Clinically Relevant Subtypes of Glioblastoma Characterized by Abnormalities in PDGFRA, IDH1, EGFR, and NF1. Cancer Cell.

[3] Hanahan D., Weinberg R.A. (2011). Hallmarks of cancer: The next generation. Cell.

[4] Stupp R., Hegi M.E., Mason W.P., van den Bent M.J., Taphoorn M.J., Janzer R.C., Ludwin S.K., Allgeier A., Fisher B., Belanger K. (2009). Effects of radiotherapy with concomitant and adjuvant temozolomide versus radiotherapy alone on survival in glioblastoma in a randomised phase III study: 5-year analysis of the EORTC-NCIC trial. Lancet Oncol..

[5] Cha J., Kang S.G., Kim P. (2016). Strategies of Mesenchymal Invasion of Patient-derived Brain Tumors: Microenvironmental Adaptation. Sci. Rep..

[6] Keime-Guibert F., Chinot O., Taillandier L., Cartalat-Carel S., Frenay M., Kantor G., Guillamo J.S., Jadaud E., Colin P., Bondiau P.Y. (2007). Radiotherapy for glioblastoma in the elderly. N. Engl. J. Med..

[7] Lang F.F., Gilbert M.R. (2006). Diffusely infiltrative low-grade gliomas in adults. J. Clin. Oncol..

[8] Laperriere N., Zuraw L., Cairncross G. (2002). Radiotherapy for newly diagnosed malignant glioma in adults: A systematic review. Radiother. Oncol..

[9] Yaşargil M.G., von Ammon K., Cavazos E., Doczi T., Reeves J.D., Roth P. (1992). Tumours of the limbic and paralimbic systems. Acta Neurochir..

[10] Schramm J., Aliashkevich A.F. (2007). Surgery for temporal mediobasal tumors: Experience based on a series of 235 patients. Neurosurgery.

[11] Scherer H.J. (1938). Structural Development in Gliomas. Am. J. Cancer.

[12] Farin A., Suzuki S.O., Weiker M., Goldman J.E., Bruce J.N., Canoll P. (2006). Transplanted glioma cells migrate and proliferate on host brain vasculature: A dynamic analysis. Glia.

[13] De Gooijer M.C., Guillén Navarro M., Bernards R., Wurdinger T., van Tellingen O. (2018). An Experimenter’s Guide to Glioblastoma Invasion Pathways. Trends Mol. Med..

[14] Cuddapah V.A., Robel S., Watkins S., Sontheimer H. (2014). A neurocentric perspective on glioma invasion. Nat. Rev. Neurosci..

[15] Montana V., Sontheimer H. (2011). Bradykinin promotes the Chemotactic invasion of primary brain tumors. J. Neurosci..

[16] Diksin M., Smith S.J., Rahman R. (2017). The molecular and phenotypic basis of the glioma invasive perivascular niche. Int. J. Mol. Sci..

[17] Watkins S., Robel S., Kimbrough I.F., Robert S.M., Ellis-Davies G., Sontheimer H. (2014). Disruption of astrocyte–vascular coupling and the blood–brain barrier by invading glioma cells. Nat. Commun..

[18] Mughal A.A., Zhang L., Fayzullin A., Server A., Li Y., Wu Y., Glass R., Meling T., Langmoen I.A., Leergaard T.B. (2018). Patterns of Invasive Growth in Malignant Gliomas—The Hippocampus Emerges as an Invasion-Spared Brain Region. Neoplasia.

[19] Wang J., Yi L., Kang Q., Zhou J., Chen T., Hugnot J., Yu S. (2022). Glioma invasion along white matter tracts: A dilemma for neurosurgeons. Cancer Lett..

[20] Louis D.N., Perry A., Reifenberger G., von Deimling A., Figarella-Branger D., Cavenee W.K., Ohgaki H., Wiestler O.D., Kleihues P., Ellison D.W. (2016). The 2016 World Health Organization Classification of Tumors of the Central Nervous System: A summary. Acta Neuropathol..

[21] Chédotal A., Kerjan G., Moreau-Fauvarque C. (2005). The brain within the tumor: New roles for axon guidance molecules in cancers. Cell Death Differ..

[22] Claes A., Idema A.J., Wesseling P. (2007). Diffuse glioma growth: A guerilla war. Acta Neuropathol..

[23] Coniglio S.J., Eugenin E., Dobrenis K., Stanley E.R., West B.L., Symons M.H., Segall J.E. (2012). Microglial stimulation of glioblastoma invasion involves epidermal growth factor receptor (EGFR) and colony stimulating factor 1 receptor (CSF-1R) signaling. Mol. Med..

[24] Kunkel P., Müller S., Schirmacher P., Stavrou D., Fillbrandt R., Westphal M., Lamszus K. (2004). Expression and localization of scatter factor/hepatocyte growth factor in human astrocytomas. Neuro. Oncol..

[25] Gatenby R.A., Gillies R.J. (2004). Why do cancers have high aerobic glycolysis?. Nat. Rev. Cancer.

[26] Gatenby R.A., Gawlinski E.T., Gmitro A.F., Kaylor B., Gillies R.J. (2006). Acid-mediated tumor invasion: A multidisciplinary study. Cancer Res..

[27] Xie Q., Mittal S., Berens M.E. (2014). Targeting adaptive glioblastoma: An overview of proliferation and invasion. Neuro. Oncol..

[28] Ulrich T.A., De Juan Pardo E.M., Kumar S. (2009). The mechanical rigidity of the extracellular matrix regulates the structure, motility, and proliferation of glioma cells. Cancer Res..

[29] Byrne K.M., Monsefi N., Dawson J.C., Degasperi A., Bukowski-Wills J.C., Volinsky N., Dobrzyński M., Birtwistle M.R., Tsyganov M.A., Kiyatkin A. (2016). Bistability in the Rac1, PAK, and RhoA Signaling Network Drives Actin Cytoskeleton Dynamics and Cell Motility Switches. Cell Syst..

[30] Liu Y.J., Le Berre M., Lautenschlaeger F., Maiuri P., Callan-Jones A., Heuzé M., Takaki T., Voituriez R., Piel M. (2015). Confinement and low adhesion induce fast amoeboid migration of slow mesenchymal cells. Cell.

[31] Paňková K., Rösel D., Novotný M., Brábek J. (2010). The molecular mechanisms of transition between mesenchymal and amoeboid invasiveness in tumor cells. Cell. Mol. Life Sci..

[32] O’Neill G.M., Zhong J., Paul A., Kellie S.J. (2010). Mesenchymal migration as a therapeutic target in glioblastoma. J. Oncol..

[33] Maaser K., Wolf K., Klein C.E., Niggemann B., Zänker K.S., Bröcker E.B., Friedl P. (1999). Functional hierarchy of simultaneously expressed adhesion receptors: Integrin α2β1 but not CD44 mediates MV3 melanoma cell migration and matrix reorganization within three-dimensional hyaluronan-containing collagen matrices. Mol. Biol. Cell.

[34] Nobes C.D., Hall A. (1995). Rho, Rac, and Cdc42 GTPases regulate the assembly of multimolecular focal complexes associated with actin stress fibers, lamellipodia, and filopodia. Cell.

[35] Demuth T., Berens M.E. (2004). Molecular mechanisms of glioma cell migration and invasion. J. Neurooncol..

[36] Osswald M., Jung E., Sahm F., Solecki G., Venkataramani V., Blaes J., Weil S., Horstmann H., Wiestler B., Syed M. (2015). Brain tumour cells interconnect to a functional and resistant network. Nature.

[37] Bastola S., Pavlyukov M.S., Yamashita D., Ghosh S., Cho H., Kagaya N., Zhang Z., Minata M., Lee Y., Sadahiro H. (2020). Glioma-initiating cells at tumor edge gain signals from tumor core cells to promote their malignancy. Nat. Commun..

[38] Alieva M., Leidgens V., Riemenschneider M.J., Klein C.A., Hau P., van Rheenen J. (2019). Intravital imaging of glioma border morphology reveals distinctive cellular dynamics and contribution to tumor cell invasion. Sci. Rep..

[39] Friedl P., Alexander S. (2011). Cancer invasion and the microenvironment: Plasticity and reciprocity. Cell.

[40] Virga J., Szivos L., Hortobágyi T., Chalsaraei M.K., Zahuczky G., Steiner L., Tóth J., Reményi-Puskár J., Bognár L., Klekner A. (2019). Extracellular matrix differences in glioblastoma patients with different prognoses. Oncol. Lett..

[41] Novak U., Kaye A.H. (2000). Extracellular matrix and the brain: Components and function. J. Clin. Neurosci..

[42] Goldbrunner R.H., Bernstein J.J., Tonn J.C. (1999). Cell-extracellular matrix interaction in glioma invasion. Acta Neurochir..

[43] Tysnes B.B., Mahesparan R., Thorsen F., Haugland H.K., Porwol T., Enger P.O., Lund-Johansen M., Bjerkvig R. (1999). Laminin expression by glial fibrillary acidic protein positive cells in human gliomas. Int. J. Dev. Neurosci..

[44] Read R.M.T., Kai M.L., Skaftnesmo O., Bjerkvig R., Engebraaten O. (2003). Expression of extracellular matrix components in a highly infiltrative in vivo glioma model. Acta Neuropathol..

[45] Bjerkvig R., Laerum O.D., Rucklidge G.J. (1989). Immunocytochemical characterization of extracellular matrix proteins expressed by cultured glioma cells. Cancer Res..

[46] Baldwin J.R., McKeever P.E., Booker T.R. (1985). Products of cultured neuroglial cells: II. The production of fibronectin by C6 glioma cells. Neurochem. Res..

[47] Savaraj N., Wu C., Landy H., Wangpaijit M., Wei Y., Kuo M.T., Robles C., Furst A.J., Lampidis T., Feun L. (2005). Procollagen alpha 1 type I: A potential aide in histopathological grading of glioma. Cancer Investig..

[48] Paulus W., Roggendorf W., Schuppan D. (1988). Immunohistochemical investigation of collagen subtypes in human glioblastomas. Virchows Arch. A Pathol. Anat. Histopathol..

[49] Han J., Daniel J.C., Pappas G.D. (1995). Invasion in Brain Tissue Cultures. Cancer.

[50] De Clerck Y.A., Shimada H., Gonzalez-Gomez I., Raffel C. (1993). Tumoral invasion in the central nervous system. J. Neurooncol..

[51] Diao W., Tong X., Yang C., Zhang F., Bao C., Chen H., Liu L., Li M., Ye F., Fan Q. (2019). Behaviors of Glioblastoma Cells in in Vitro Microenvironments. Sci. Rep..

[52] Giese A., Westphal M. (1996). Glioma invasion in the central nervous system. Neurosurgery.

[53] Mechanism A. (1991). Glioblastoma Expression of Vitronectin and the av/83 Integrin. Adhesion mechanism for transformed glial cells. J. Clin. Investig..

[54] Uhm J.H., Dooley N.P., Kyritsis A.P., Rao J.S., Gladson C.L. (1999). Vitronectin, a glioma-derived extracellular matrix protein, protects tumor cells from apoptotic death. Clin. Cancer Res..

[55] Higuchi M., Ohnishi T., Arita N., Hiraga S., Hayakawa T. (1993). Expression of tenascin in human gliomas: Its relation to histological malignancy, tumor dedifferentiation and angiogenesis. Acta Neuropathol..

[56] Bourdon M.A., Wikstrand C.J., Furthmayr H., Matthews T.J., Bigner D.D. (1983). Human Glioma-Mesenchymal Extracellular Matrix Antigen Defined by Monoclonal Antibody Human Glioma-Mesenchymal Monoclonal Antibody Extracellular Matrix Antigen Defined by. Cancer Res..

[57] McComb R.D., Bigner D.D. (1985). Immunolocalization of Laminin in Neoplasms of the Central and Peripheral Nervous Systems. J. Neuropathol. Exp. Neurol..

[58] Marienhagen K., Bjerkvig R. (1993). Migratory Pattern of Fetal Rat Brain Cells and Human Glioma Cells in the Adult Rat Brain. Cancer Res..

[59] Gkretsi V., Stylianopoulos T. (2018). Cell Adhesion and Matrix Stiffness: Coordinating Cancer Cell Invasion and Metastasis. Front. Oncol..

[60] Nagano O., Saya H. (2004). Mechanism and biological significance of CD44 cleavage. Cancer Sci..

[61] Ranuncolo S.M., Ladeda V., Specterman S. (2002). CD44 Expression in Human Gliomas. J. Surg. Oncol..

[62] Okamoto I., Kawano Y., Matsumoto M., Suga M., Kaibuchi K., Ando M., Saya H. (1999). Regulated CD44 Cleavage under the Control of Protein Kinase C, Calcium Influx, and the Rho Family of Small G Proteins. J. Biol. Chem..

[63] Aruffo A., Stamenkovic I., Melnick M., Underhill C.B., Seed B. (1990). CD44 is the principal cell surface receptor for hyaluronate. Cell.

[64] Bellail A.C., Hunter S.B., Brat D.J., Tan C., Van Meir E.G. (2004). Microregional extracellular matrix heterogeneity in brain modulates glioma cell invasion. Int. J. Biochem. Cell Biol..

[65] Edvardsen K., Chent W., Rucklidgef G., Walsh F.S., Obrinkt B., Bock E. (2000). Transmembrane neural cell-adhesion molecule (NCAM), but not secretion of matrix metalloproteinases. Proc. Natl. Acad. Sci. USA.

[66] Prag S., Lepekhin E.A., Kolkova K., Hartmann-petersen R., Kawa A., Walmod P.S., Belman V., Gallagher H.C., Berezin V., Bock E. (2002). NCAM regulates cell motility. J. Cell Sci..

[67] Cavallaro U., Christofori G. (2004). Cell adhesion and signalling by cadherins and Ig-CAMs in cancer. Nat. Rev. Cancer.

[68] Perego C., Vanoni C., Massari S., Raimondi A., Pola S., Cattaneo M.G., Francolini M., Vicentini L.M., Pietrini G. (2002). Invasive behaviour of glioblastoma cell lines is associated with altered organisation of the cadherin- catenin adhesion system. J. Cell Sci..

[69] Wenk M.B., Midwood K.S., Schwarzbauer J.E. (2000). Tenascin-C Suppresses Rho Activation. J. Cell Biol..

[70] Ellert-Miklaszewska A., Poleszak K., Pasierbinska M., Kaminska B. (2020). Integrin signaling in glioma pathogenesis: From biology to therapy. Int. J. Mol. Sci..

[71] Horwitz A.F. (1997). Integrins and health. Sci. Am..

[72] Biology C., Hospital H. (1998). Stimulation of extracellular matrix components in the normal. Int. J. Cancer.

[73] Paulus W., Baur I., Schuppan D., Roggendorf W. (1993). Characterization of integrin receptors in normal and neoplastic human brain. Am. J. Pathol..

[74] Gingras M.C., Roussel E., Bruner J.M., Branch C.D., Moser R.P. (1995). Comparison of cell adhesion molecule expression between glioblastoma multiforme and autologous normal brain tissue. J. Neuroimmunol..

[75] Cary L.A., Guan J.L. (1999). Focal adhesion kinase in integrin-mediated signaling. Front. Biosci..

[76] Cox E.A., Huttenlocher A. (1998). Regulation of Integrin-Mediated Adhesion During Cell Migration. Microsc. Res. Tech..

[77] Munson J., Bonner M., Fried L., Hofmekler J., Arbiser J., Bellamkonda R. (2013). Identifying new small molecule anti-invasive compounds for glioma treatment. Cell Cycle.

[78] Hauck C.R., Hsia D.A., Schlaepfer D.D. (2002). The Focal Adhesion Kinase—A Regulator of Cell Migration and Invasion Structural Characteristics of FAK-like Protein Tyrosine Kinases Focal adhesion kinase (FAK) 1 together with Pyk2 (1) form a subfamily of FAK-like protein-tyrosine kinases (PTKs). FAK. IUBMB Life.

[79] Owens L.V., Xu L.H., Craven R.J., Dent G.A., Weiner T.M., Kornberg L., Liu E.T., Cance W.G. (1995). Overexpression of the Focal Adhesion Kinase (p125FAK) in Invasive Human Tumors. Cancer Res..

[80] Zagzag D., Friedlander D.R., Margolis B., Grumet M., Semenza G.L., Zhong H., Simons J.W., Holash J., Wiegand S.J., Yancopoulos G.D. (2000). Molecular events implicated in brain tumor angiogenesis and invasion. Pediatr. Neurosurg..

[81] Wilkins-port C.E., Freytag J., Higgins S.P., Higgins P.J. (2010). PAI-1: A Multifunctional SERPIN with Complex Roles in Cell Signaling and Migration. Cell Commun. Insights.

[82] Deryugina E.I., Ratnikov B., Monosov E., Postnova T.I., Discipio R., Smith J.W., Strongin A.Y. (2001). MT1-MMP Initiates Activation of pro-MMP-2 and Integrin αvβ3 Promotes Maturation of MMP-2 in Breast Carcinoma Cells. Exp. Cell Res..

[83] Rao J.S., Steck P.A., Tofilon P., Boyd D., Aliosman F., Stetlerstevenson W.G., Liotta L.A., Sawaya R. (1994). Role of Plasminogen-Activator and of 92-Kda Type-Iv Collagenase in Glioblastoma Invasion Using an in-Vitro Matrigel Model. J. Neurooncol..

[84] Kesanakurti D., Chetty C., Dinh D.H., Gujrati M., Rao J.S. (2013). Role of MMP-2 in the regulation of IL-6/Stat3 survival signaling via interaction with α5β1 integrin in glioma. Oncogene.

[85] Raithatha S.A., Muzik H., Muzik H., Rnewcastle N.B., Johnston R.N., Edwards D.R., Forsyth P.A. (2000). Localization of gelatinase-A and gelatinase-B mRNA and protein in human gliomas. Neuro-Oncology.

[86] Nakada M., Miyamori H., Kita D. (2005). Human glioblastomas overexpress ADAMTS-5 that degrades brevican. Acta Neuropathol..

[87] Held-feindt J., Paredes E.B., Bl U., Seidenbecher C., Stark A.M., Mehdorn H.M., Mentlein R. (2006). Matrix-degrading proteases ADAMTS4 and ADAMTS5 (disintegrins and metalloproteinases with thrombospondin motifs 4 and 5) are expressed in human glioblastomas. Int. J. Cancer.

[88] Blasi F., Carmeliet P. (2002). uPAR: A versatile signalling orchestrator. Nat. Rev. Mol. Cell Biol..

[89] Shen L.J., Wang S.Y., Xie G.F., Zeng Q., Chen C., Dong A.N., Huang Z.M. (2015). Subdivision of M category for nasopharyngeal carcinoma with synchronous metastasis: Time to expand the M categorization system. Chin. J. Cancer.

[90] Geis T., Döring C., Popp R., Grossmann N., Fleming I., Hansmann M., Dehne N., Brüne B. (2014). HIF-2alpha-dependent PAI-1 induction contributes to angiogenesis in hepatocellular carcinoma. Exp. Cell Res..

[91] Colin C., Voutsinos-Porche B., Nanni I., Fina F., Metellus P., Intagliata D., Baeza N., Bouvier C., Delfino C., Loundou A. (2009). High expression of cathepsin B and plasminogen activator inhibitor type-1 are strong predictors of survival in glioblastomas. Acta Neuropathol..

[92] Iwadate Y., Hayama M., Adachi A., Matsutani T. (2008). High Serum Level of Plasminogen Activator Inhibitor-1 Predicts Histological Grade of Intracerebral Gliomas. Anticancer Res..

[93] Ford H., Hospital H.F., State W. (1995). Immunolocalization of cathepsin B in human glioma: Implications for tumor invasion and angiogenesis. J. Neurosurg..

[94] Gondi C.S., Lakka S.S., Yanamandra N., Olivero W.C., Dinh D.H., Gujrati M., Tung C.H., Weissleder R., Rao J.S. (2004). Advances in Brief Adenovirus-Mediated Expression of Antisense Urokinase Plasminogen Activator Receptor and Antisense Cathepsin B Inhibits Tumor Growth, Invasion, and Angiogenesis in Gliomas. Cancer Res..

[95] Demchik L.L., Sameni M., Nelson K., Mikkelsen T., Sloane B.F. (1999). Cathepsin B and glioma invasion. Int. J. Dev. Neurosci..

[96] Sivaparvathi M., Sawaya R., Wu Wang S., Rayford A., Yamamoto M., Liottat L.A., Nicolson G.L., Rao J.S. (1995). Overexpression and localization of cathepsin B during the progression of human gliomas. Clin. Exp. Metastasis.

[97] Tysnes B.B., Mahesparan R. (2001). Biological mechanisms of glioma invasion and potential therapeutic targets. J. Neurooncol..

[98] Nicholson-Dykstra S., Higgs H.N., Harris E.S. (2005). Actin dynamics: Growth from dendritic branches. Curr. Biol..

[99] Whale A., Hashim F.N., Fram S., Jones G.E., Wells C.M. (2011). Signalling to cancer cell invasion through PAK family kinases. Front. Biosci..

[100] Yamaguchi H., Oikawa T. (2010). Membrane lipids in invadopodia and podosomes: Key structures for cancer invasion and metastasis. Oncotarget.

[101] Yamaguchi H., Condeelis J. (2007). Regulation of the actin cytoskeleton in cancer cell migration and invasion. Biochim. Biophys. Acta-Mol. Cell Res..

[102] Wang H., Han M., Whetsell W., Wang J., Rich J., Hallahan D., Han Z. (2014). Tax-interacting protein 1 coordinates the spatiotemporal activation of Rho GTPases and regulates the infiltrative growth of human glioblastoma. Oncogene.

[103] Lamszus K., Schmidt N.O., Jin L., Laterra J., Zagzag D., Way D., Witte M., Weinand M., Goldberg I.D., Westphal M. (1998). Scatter factor promotes motility of human glioma and neuromicrovascular endothelial cells. Int. J. Cancer.

[104] Watanabe K., Tachibana O., Sato K., Yonekawa Y., Kleihues P., Ohgaki H. (1996). Overexpression of the EGF receptor and p53 mutations are mutually exclusive in the evolution of primary and secondary glioblastomas. Brain Pathol..

[105] Gumbiner B.M. (2005). Regulation of cadherin-mediated adhesion in morphogenesis. Nat. Rev. Mol. Cell Biol..

[106] Lund-johansen M., Bjcrkvig R., Humphrey P.A., Bigner S.H., Bigner D.D., Laerum O. (1990). Effect of Epidermal Growth Factor on Glioma Cell Growth, Migration, and Invasion in Vitro. Cancer Res..

[107] Coniglio S.J., Segall J.E. (2021). Microglial-stimulation of glioma invasion involves the EGFR ligand amphiregulin. PLoS ONE.

[108] Joseph J.V., Magaut C.R., Storevik S., Geraldo L.H., Mathivet T., Latif M.A., Rudewicz J., Guyon J., Gambaretti M., Haukas F. (2021). TGF-β promotes microtube formation in glioblastoma through Thrombospondin 1. Neuro. Oncol..

[109] Hemler M.E. (1998). Integrin associated proteins. Curr. Opin. Cell Biol..

[110] Burridge K., Chrzanowska-Wodnicka M. (1996). Focal adhesions, contractility, and signaling. Annu. Rev. Cell Dev. Biol..

[111] Cukierman E., Cukierman E., Pankov R., Stevens D.R. (2013). Taking Cell-Matrix Adhesions to the Third Dimension. Science.

[112] Rabinovitz I., Mercurio A.M. (1997). The Integrin α6β4 Functions in Carcinoma Cell Migration on Laminin-1 by Mediating the Formation and Stabilization of Actin-containing Motility Structures. J. Cell Biol..

[113] De Semir D., Bezrookove V., Nosrati M., Scanlon K.R., Singer E., Judkins J., Rieken C., Wu C., Shen J., Schmudermayer C. (2020). PHIP drives glioblastoma motility and invasion by regulating the focal adhesion complex. Proc. Natl. Acad. Sci. USA.

[114] Porčnik A., Novak M., Breznik B., Majc B., Hrastar B., Šamec N., Zottel A., Jovčevska I. (2021). TRIM28 Selective Nanobody Reduces Glioblastoma Stem Cell Invasion. Molecules.

[115] Chen L., Zhu M., Yu S., Hai L., Zhang L., Zhang C., Zhao P., Zhou H., Wang S., Yang X. (2020). Arg kinase mediates CXCL12/CXCR4-induced invadopodia formation and invasion of glioma cells. Exp. Cell Res..

[116] Yi L., Zhou X., Li T., Liu P., Hai L., Tong L., Ma H., Tao Z., Xie Y., Zhang C. (2019). Notch1 signaling pathway promotes invasion, self-renewal and growth of glioma initiating cells via modulating chemokine system CXCL12/CXCR4. J. Exp. Clin. Cancer Res..

[117] Litan A., Langhans S.A. (2015). Cancer as a channelopathy: Ion channels and pumps in tumor development and progression. Front. Cell. Neurosci..

[118] Caramia M., Sforna L., Franciolini F., Catacuzzeno L. (2019). The volume-regulated anion channel in glioblastoma. Cancers.

[119] Sun H., Long S., Wu B., Liu J., Li G. (2020). NKCC1 involvement in the epithelial-to-mesenchymal transition is a prognostic biomarker in gliomas. PeerJ.

[120] Zou W. (2019). Potassium Channel and Glioma. Biomed. J. Sci. Tech. Res..

[121] Catacuzzeno L., Franciolini F. (2018). Role of KCa3.1 channels in modulating Ca^2+^ oscillations during glioblastoma cell migration and invasion. Int. J. Mol. Sci..

[122] Thuringer D., Chanteloup G., Boucher J., Pernet N., Boudesco C., Jego G., Chatelier A., Bois P., Gobbo J., Cronier L. (2017). Modulation of the inwardly rectifying potassium channel Kir4.1 by the pro-invasive miR-5096 in glioblastoma cells. Oncotarget.

[123] Brandalise F., Ratto D., Leone R., Olivero F., Roda E., Locatelli C.A., Grazia Bottone M., Rossi P. (2020). Deeper and Deeper on the Role of BK and Kir4.1 Channels in Glioblastoma Invasiveness: A Novel Summative Mechanism?. Front. Neurosci..

[124] Liu M., Inoue K., Leng T., Guo S., Xiong Z.-G. (2014). TRPM7 channels regulate glioma stem cell through STAT3 and Notch signaling pathways. Cell. Signal..

[125] Bao M.H., Lv Q.L., Szeto V., Wong R., Zhu S.Z., Zhang Y.Y., Feng Z.P., Sun H.S. (2020). TRPM2-AS inhibits the growth, migration, and invasion of gliomas through JNK, c-Jun, and RGS4. J. Cell. Physiol..

[126] Wong R., Gong H., Alanazi R., Bondoc A., Luck A., Sabha N., Horgen F.D., Fleig A., Rutka J.T., Feng Z.P. (2020). Inhibition of TRPM7 with waixenicin A reduces glioblastoma cellular functions. Cell Calcium.

[127] Shah S., Chu Y., Cegielski V., Chu X.P. (2021). Acid-Sensing Ion Channel 1 Contributes to Weak Acid-Induced Migration of Human Malignant Glioma Cells. Front. Physiol..

[128] Kalluri R., Weinberg R.A. (2009). The basics of epithelial-mesenchymal transition. J. Clin. Investig..

[129] Iser I.C., Pereira M.B., Lenz G., Wink M.R. (2017). The Epithelial-to-Mesenchymal Transition-Like Process in Glioblastoma: An Updated Systematic Review and In Silico Investigation. Med. Res. Rev..

[130] Kahlert U.D., Joseph J.V., Kruyt F.A.E. (2017). EMT- and MET-related processes in nonepithelial tumors: Importance for disease progression, prognosis, and therapeutic opportunities. Mol. Oncol..

[131] Thiery J.P., Acloque H., Huang R.Y.J., Nieto M.A. (2009). Epithelial-Mesenchymal Transitions in Development and Disease. Cell.

[132] Bakir B., Chiarella A.M., Pitarresi J.R., Rustgi A.K. (2020). EMT, MET, Plasticity, and Tumor Metastasis. Trends Cell Biol..

[133] Iwadate Y. (2016). Epithelial-mesenchymal transition in glioblastoma progression. Oncol. Lett..

[134] Xie Q., Thompson R., Hardy K., DeCamp L., Berghuis B., Sigler R., Knudsen B., Cottingham S., Zhao P., Dykema K. (2008). A highly invasive human glioblastoma pre-clinical model for testing therapeutics. J. Transl. Med..

[135] Siebzehnrubl F.A., Silver D.J., Tugertimur B., Deleyrolle L.P., Siebzehnrubl D., Sarkisian M.R., Devers K.G., Yachnis A.T., Kupper M.D., Neal D. (2013). The ZEB1 pathway links glioblastoma initiation, invasion and chemoresistance. EMBO Mol. Med..

[136] Jia Y., Feng Q., Tang B., Luo X., Yang Q., Yang H., Li Q. (2021). Decorin Suppresses Invasion and EMT Phenotype of Glioma by Inducing Autophagy via c-Met/Akt/mTOR Axis. Front. Oncol..

[137] Wang Y., Shi J., Chai K., Ying X., Zhou B. (2014). The Role of Snail in EMT and Tumorigenesis. Curr. Cancer Drug Targets.

[138] Myung J.K., Choi S.A., Kim S.K., Wang K.C., Park S.H. (2014). Snail plays an oncogenic role in glioblastoma by promoting epithelial mesenchymal transition. Int. J. Clin. Exp. Pathol..

[139] Yang H.W., Menon L.G., Black P.M., Carroll R.S., Johnson M.D. (2010). SNAI2/Slug promotes growth and invasion in human gliomas. BMC Cancer.

[140] Lin J., Jiang L., Wang X., Wei W., Song C., Cui Y., Wu X., Qiu G. (2021). P4HA2 Promotes Epithelial-to-Mesenchymal Transition and Glioma Malignancy through the Collagen-Dependent PI3K/AKT Pathway. J. Oncol..

[141] Wu Y., Zhang X., Wang J., Ji R., Zhang L., Qin J., Tian M., Jin G., Zhang X. (2021). P4HA2 promotes cell proliferation and migration in glioblastoma. Oncol. Lett..

[142] Storci G., Sansone P., Mari S., D’Uva G., Tavolari S., Guarnieri T., Taffurelli M., Ceccarelli C., Santini D., Chieco P. (2010). TNFalpha up-regulates SLUG via the NF-kappaB/HIF1alpha axis, which imparts breast cancer cells with a stem cell-like phenotype. J. Cell. Physiol..

[143] Kim J., Kong J., Chang H., Kim H., Kim A. (2016). EGF induces epithelial-mesenchymal transition through phospho-Smad2/3-Snail signaling pathway in breast cancer cells. Oncotarget.

[144] Mariani L., McDonough W.S., Hoelzinger D.B., Beaudry C., Kacsmarek E., Coons S.W., Giese A., Moghaddam M., Seiler R.W., Berens M.E. (2001). Identification and validation of *P311* as a glioblastoma invasion gene using laser capture microdissection. Cancer Res..

[145] Hoelzinger D.B., Mariani L., Weis J., Woyke T., Berens T.J., McDonough W., Sloan A., Coons S.W., Berens M.E. (2006). Gene Expression Profile of Glioblastoma Multiforme Invasive Phenotype Points to New Therapeutic Targets. Neoplasia.

[146] Demuth T., Reavie L.B., Rennert J.L., Nakada M., Nakada S., Hoelzinger D.B., Beaudry C.E., Henrichs A.N., Anderson E.M., Berens M.E. (2007). MAP-ing glioma invasion: Mitogen-activated protein kinase kinase 3 and p38 drive glioma invasion and progression and predict patient survival. Mol. Cancer Ther..

[147] Demuth T., Rennert J.L., Hoelzinger D.B., Reavie L.B., Nakada M., Beaudry C., Nakada S., Anderson E.M., Henrichs A.N., McDonough W.S. (2008). Glioma cells on the run—The migratory transcriptome of 10 human glioma cell lines. BMC Genom..

[148] Krieger T.G., Tirier S.M., Park J., Jechow K., Eisemann T., Peterziel H., Angel P., Eils R., Conrad C. (2020). Modeling glioblastoma invasion using human brain organoids and single-cell transcriptomics. Neuro. Oncol..

[149] Seker F., Cingoz A., Sur-Erdem İ., Erguder N., Erkent A., Uyulur F., Selvan M.E., Gümüş Z.H., Gönen M., Bayraktar H. (2019). Identification of SERPINE1 as a regulator of glioblastoma cell dispersal with transcriptome profiling. Cancers.

[150] Zhou K., Zhang C., Yao H., Zhang X., Zhou Y., Che Y., Huang Y. (2018). Knockdown of long non-coding RNA NEAT1 inhibits glioma cell migration and invasion via modulation of SOX2 targeted by miR-132. Mol. Cancer.

[151] Li N., Zhang Y., Sidlauskas K., Ellis M., Evans I., Frankel P., Lau J., El-Hassan T., Guglielmi L., Broni J. (2018). Inhibition of GPR158 by microRNA-449a suppresses neural lineage of glioma stem/progenitor cells and correlates with higher glioma grades. Oncogene.

[152] Haas T.L., Sciuto M.R., Brunetto L., Valvo C., Signore M., Fiori M.E., di Martino S., Giannetti S., Morgante L., Boe A. (2017). Integrin α7 Is a Functional Marker and Potential Therapeutic Target in Glioblastoma. Cell Stem Cell.

[153] Mallawaaratchy D.M., Buckland M.E., McDonald K.L., Li C.C.Y., Ly L., Sykes E.K., Christopherson R.I., Kaufman K.L. (2015). Membrane Proteome Analysis of Glioblastoma Cell Invasion. J. Neuropathol. Exp. Neurol..

[154] Pei J., Moon K.-S., Pan S., Lee K.-H., Ryu H.-H., Jung T.-Y., Kim I.-Y., Jang W.-Y., Jung C.-H., Jung S. (2014). Proteomic Analysis between U87MG and U343MG-A Cell Lines: Searching for Candidate Proteins for Glioma Invasion. Brain Tumor Res. Treat..

[155] Bagci T., Wu J.K., Pfannl R., Ilag L.L., Jay D.G. (2009). Autocrine semaphorin 3A signaling promotes glioblastoma dispersal. Oncogene.

[156] Sloan K.E., Eustace B.K., Stewart J.K., Zehetmeier C., Torella C., Simeone M., Roy J.E., Unger C., Louis D.N., Ilag L.L. (2004). CD155/PVR plays a key role in cell motility during tumor cell invasion and migration. BMC Cancer.

[157] Jarzynka M.J., Hu B., Hui K.M., Bar-Joseph I., Gu W., Hirose T., Haney L.B., Ravichandran K.S., Nishikawa R., Cheng S.Y. (2007). ELMO1 and Dock180, a bipartite Rac1 guanine nucleotide exchange factor, promote human glioma cell invasion. Cancer Res..

[158] Goplen D., Wang J., Enger P., Tysnes B.B., Terzis A.J.A., Laerum O.D., Bjerkvig R. (2006). Protein disulfide isomerase expression is related to the invasive properties of malignant glioma. Cancer Res..

[159] He L., Yang H., Zhu X.-L., Zhang Y., Kun L. (2021). Knockdown of long non-coding RNA SLC8A1-AS1 attenuates cell invasion and migration in glioma via suppression of Wnt/β-catenin signaling pathways. Brain Res. Bull..

[160] Diao Y., Jin B., Huang L., Zhou W. (2018). MiR-129-5p inhibits glioma cell progression in vitro and in vivo by targeting TGIF2. J. Cell. Mol. Med..

[161] Xu Z., Chen Q., Zeng X., Li M., Liao J. (2021). Lnc-NLC1-C inhibits migration, invasion and autophagy of glioma cells by targeting miR-383 and regulating PRDX-3 expression. Oncol. Lett..

[162] Lin Y., Wei L., Hu B., Zhang J., Wei J., Qian Z., Zou D. (2021). RBM8A Promotes Glioblastoma Growth and Invasion Through the Notch/STAT3 Pathway. Front. Oncol..

[163] Formolo C.A., Williams R., Gordish-Dressman H., MacDonald T.J., Lee N.H., Hathout Y. (2011). Secretome Signature of Invasive Glioblastoma Multiforme. J. Proteome Res..

[164] Dzikowski L., Mirzaei R., Sarkar S., Kumar M., Bose P., Bellail A., Hao C., Yong V.W. (2021). Fibrinogen in the glioblastoma microenvironment contributes to the invasiveness of brain tumor-initiating cells. Brain Pathol..

[165] Gritsenko P., Leenders W., Friedl P. (2017). Recapitulating in vivo-like plasticity of glioma cell invasion along blood vessels and in astrocyte-rich stroma. Histochem. Cell Biol..

[166] Palmieri G., Cofano F., Salvati L.F., Monticelli M., Zeppa P., Di Perna G., Melcarne A., Altieri R., La Rocca G., Sabatino G. (2021). Fluorescence-Guided Surgery for High-Grade Gliomas: State of the Art and New Perspectives. Technol. Cancer Res. Treat..

[167] Pekmezci M., Morshed R.A., Chunduru P., Pandian B., Young J., Villanueva-Meyer J.E., Tihan T., Sloan E.A., Aghi M.K., Molinaro A.M. (2021). Detection of glioma infiltration at the tumor margin using quantitative stimulated Raman scattering histology. Sci. Rep..

[168] Castro L.N.G., Tirosh I., Suvà M.L. (2021). Decoding cancer biology one cell at a time. Cancer Discov..

[169] Patel A.P., Tirosh I., Trombetta J.J., Shalek A.K., Gillespie S.M., Wakimoto H., Cahill D.P., Nahed B.V., Curry W.T., Martuza R.L. (2014). Single-cell RNA-seq highlights intratumoral heterogeneity in primary glioblastoma. Science.

[170] Zhang J., Stevens M.F.G., Bradshaw T.D. (2011). Temozolomide: Mechanisms of Action, Repair and Resistance. Curr. Mol. Pharmacol..

[171] Messaoudi K., Clavreul A., Lagarce F. (2015). Toward an effective strategy in glioblastoma treatment. Part I: Resistance mechanisms and strategies to overcome resistance of glioblastoma to temozolomide. Drug Discov. Today.

[172] Shen W., Hu J.A., Zheng J.S. (2014). Mechanism of temozolomide-induced antitumour effects on glioma cells. J. Int. Med. Res..

[173] Arora A., Somasundaram K. (2019). Glioblastoma vs temozolomide: Can the red queen race be won?. Cancer Biol. Ther..

[174] Hegi M.E., Diserens A.-C., Gorlia T., Hamou M.-F., de Tribolet N., Weller M., Kros J.M., Hainfellner J.A., Mason W., Mariani L. (2005). MGMT Gene Silencing and Benefit from Temozolomide in Glioblastoma. N. Engl. J. Med..

[175] Wang H., Guo J., Wang T., Wang K., Wu Z., Sun T. (2021). Efficacy and safety of bevacizumab in the treatment of adult gliomas: A systematic review and meta-analysis. BMJ Open.

[176] Dhermain F. (2014). Radiotherapy of high-grade gliomas: Current standards and new concepts, innovations in imaging and radiotherapy, and new therapeutic approaches. Chin. J. Cancer.

[177] Brem S., Abdullah K.G., De Vleeschouwer S. (2017). Glioblastoma.

[178] Mooney J., Bernstock J.D., Ilyas A., Ibrahim A., Yamashita D., Markert J.M., Nakano I. (2019). Current Approaches and Challenges in the Molecular Therapeutic Targeting of Glioblastoma. World Neurosurg..

[179] Lu C., Shervington A. (2008). Chemoresistance in gliomas. Mol. Cell. Biochem..

[180] Ghia A.J. (2018). Fractionated Radiotherapy of Intracranial Gliomas. Prog. Neurol. Surg..

[181] Larson E.W., Peterson H.E., Lamoreaux W.T., MacKay A.R., Fairbanks R.K., Call J.A., Carlson J.D., Ling B.C., Demakas J.J., Cooke B.S. (2014). Clinical outcomes following salvage Gamma Knife radiosurgery for recurrent glioblastoma. World J. Clin. Oncol..

[182] Moghaddasi L., Bezak E. (2017). Development of an integrated Monte Carlo model for glioblastoma multiforme treated with boron neutron capture therapy. Sci. Rep..

[183] Schijns V.E.J.C., Pretto C., Devillers L., Pierre D., Hofman F.M., Chen T.C., Mespouille P., Hantos P., Glorieux P., Bota D.A. (2015). First clinical results of a personalized immunotherapeutic vaccine against recurrent, incompletely resected, treatment-resistant glioblastoma multiforme (GBM) tumors, based on combined allo- and auto-immune tumor reactivity. Vaccine.

[184] Martikainen M., Essand M. (2019). Virus-based immunotherapy of glioblastoma. Cancers.

[185] Ganipineni L.P., Danhier F., Préat V. (2018). Drug delivery challenges and future of chemotherapeutic nanomedicine for glioblastoma treatment. J. Control. Release.

[186] Van Tellingen O., Yetkin-Arik B., De Gooijer M.C., Wesseling P., Wurdinger T., De Vries H.E. (2015). Overcoming the blood-brain tumor barrier for effective glioblastoma treatment. Drug Resist. Updat..

[187] Taylor O.G., Brzozowski J.S., Skelding K.A. (2019). Glioblastoma multiforme: An overview of emerging therapeutic targets. Front. Oncol..

[188] Bagci-Onder T., Wakimoto H., Anderegg M., Cameron C., Shah K. (2011). A dual PI3K/mTOR inhibitor, PI-103, cooperates with stem cell-delivered TRAIL in experimental glioma models. Cancer Res..

[189] Stupp R., Wong E.T., Kanner A.A., Steinberg D., Engelhard H., Heidecke V., Kirson E.D., Taillibert S., Liebermann F., Dbalý V. (2012). NovoTTF-100A versus physician’s choice chemotherapy in recurrent glioblastoma: A randomised phase III trial of a novel treatment modality. Eur. J. Cancer.

[190] Stupp R., Taillibert S., Kanner A., Read W., Steinberg D.M., Lhermitte B., Toms S., Idbaih A., Ahluwalia M.S., Fink K. (2017). Effect of tumor-treating fields plus maintenance temozolomide vs maintenance temozolomide alone on survival in patients with glioblastoma a randomized clinical trial. JAMA.

[191] Ostrom Q.T., Bauchet L., Davis F.G., Deltour I., Fisher J.L., Langer C.E., Pekmezci M., Schwartzbaum J.A., Turner M.C., Walsh K.M. (2014). The epidemiology of glioma in adults: A state of the science review. Neuro. Oncol..

[192] Kirson E.D., Dbalý V., Tovaryš F., Vymazal J., Soustiel J.F., Itzhaki A., Mordechovich D., Steinberg-Shapira S., Gurvich Z., Schneiderman R. (2007). Alternating electric fields arrest cell proliferation in animal tumor models and human brain tumors. Proc. Natl. Acad. Sci. USA.

[193] Kanner A.A., Wong E.T., Villano J.L., Ram Z. (2014). Post hoc analyses of intention-to-treat population in phase III comparison of NovoTTF-100A^TM^ system versus best physician’s choice chemotherapy. Semin. Oncol..

[194] Michelhaugh S.K., Kiousis S., Michelhaugh S.A., Klinger N.V., Mittal S. Abstract 4398: In vitro Tumor Treating Fields (TTFields) alter proliferation and morphology of patient-derived high-grade meningioma cell lines. Proceedings of the AACR Annual Meeting 2018.

[195] Giladi M., Schneiderman R.S., Voloshin T., Porat Y., Munster M., Blat R., Sherbo S., Bomzon Z., Urman N., Itzhaki A. (2015). Mitotic Spindle Disruption by Alternating Electric Fields Leads to Improper Chromosome Segregation and Mitotic Catastrophe in Cancer Cells. Sci. Rep..

[196] Ornelas A.S., Porter A.B., Sharma A., Knox M.G., Marks L.A., Wingerchuk D.M., O’Carroll C.B. (2019). What is the role of tumor-Treating fields in newly diagnosed glioblastoma?. Neurologist.

[197] Guzauskas G.F., Salzberg M., Wang B.C. (2018). Estimated lifetime survival benefit of tumor treating fields and temozolomide for newly diagnosed glioblastoma patients. CNS Oncol..

[198] Mittal S., Klinger N.V., Michelhaugh S.K., Barger G.R., Pannullo S.C., Juhász C. (2018). Alternating electric tumor treating fields for treatment of glioblastoma: Rationale, preclinical, and clinical studies. J. Neurosurg..

[199] Piao Y., Liang J., Holmes L., Zurita A.J., Henry V., Heymach J.V., De Groot J.F. (2012). Glioblastoma resistance to anti-VEGF therapy is associated with myeloid cell infiltration, stem cell accumulation, and a mesenchymal phenotype. Neuro. Oncol..

[200] Gliemroth J., Feyerabend T., Gerlach C., Arnold H., Terzis A.J.A. (2003). Proliferation, migration, and invasion of human glioma cells exposed to fractionated radiotherapy in vitro. Neurosurg. Rev..

[201] Wild-bode C., Weller M., Rimner A., Dichgans J., Wick W. (2001). Sublethal Irradiation Promotes Migration and Invasiveness of Glioma Cells: Implications for Radiotherapy of Human Glioblastoma Sublethal Irradiation Promotes Migration and Invasiveness of Glioma Cells: Implications for Radiotherapy of Human Glioblastoma. Cancer Res..

[202] Giannelli G., Villa E., Lahn M. (2014). Transforming Growth Factor-β as a Therapeutic Target in Hepatocellular Carcinoma. Cancer Res..

[203] Wick A., Desjardins A., Suarez C., Forsyth P., Gueorguieva I., Burkholder T., Cleverly A.L., Estrem S.T., Wang S., Lahn M.M. (2020). Phase 1b/2a study of galunisertib, a small molecule inhibitor of transforming growth factor-beta receptor I, in combination with standard temozolomide-based radiochemotherapy in patients with newly diagnosed malignant glioma. Investig. New Drugs.

[204] Lefranc F., Le E., Kiss R., Weller M. (2018). Glioblastoma quo vadis: Will migration and invasiveness reemerge as therapeutic targets?. Cancer Treat. Rev..

[205] Weller M., Butowski N., Tran D.D., Recht L.D., Lim M., Hirte H., Ashby L., Mechtler L., Goldlust S.A., Iwamoto F. (2017). Rindopepimut with temozolomide for patients with newly diagnosed, EGFRvIII-expressing glioblastoma (ACT IV): A randomised, double-blind, international phase 3 trial. Lancet Oncol..

[206] Levin V.A., Phuphanich S., Yung W.K.A., Forsyth P.A., Del Maestro R., Perry J.R., Fuller G.N., Baillet M. (2006). Randomized, double-blind, placebo-controlled trial of marimastat in glioblastoma multiforme patients following surgery and irradiation. J. Neurooncol..

[207] Stupp R., Hegi M.E., Neyns B., Goldbrunner R., Schlegel U., Clement P.M.J., Grabenbauer G.G., Ochsenbein A.F., Simon M., Dietrich P.Y. (2010). Phase I/IIa study of cilengitide and temozolomide with concomitant radiotherapy followed by cilengitide and temozolomide maintenance therapy in patients with newly diagnosed glioblastoma. J. Clin. Oncol..

